# Intestinal Absorption Study of a Granular Form of Ferric Pyrophosphate

**DOI:** 10.3390/metabo12050463

**Published:** 2022-05-21

**Authors:** Marta Micheletto, Elisa Gaio, Erik Tedesco, Giovanni Di Maira, Etienne Mantovan, Michela Zanella, Paolo Pastore, Marco Roverso, Gabriella Favaro, Federico Benetti

**Affiliations:** 1 ECSIN-European Center for the Sustainable Impact of Nanotechnology, ECAMRICERT SRL, 35127 Padova, Italy; m.micheletto@ecamricert.com (M.M.); e.gaio@ecamricert.com (E.G.); e.tedesco@ecamricert.com (E.T.); g.dimaira@ecamricert.com (G.D.M.); e.mantovan@ecamricert.com (E.M.); m.zanella@ecamricert.com (M.Z.); 2 Department of Chemical Sciences, University of Padova, 35131 Padova, Italy; paolo.pastore@unipd.it (P.P.); marco.roverso@unipd.it (M.R.); gabriella.favaro@unipd.it (G.F.)

**Keywords:** iron, dietary supplements, intestinal absorption, delivery systems, ferritin

## Abstract

Iron deficiency is one of the most prevalent nutritional disorders worldwide. The standard treatment involves iron supplementation, but this task is challenging because of poor solubility and organoleptic issues. Moreover, the need to increase iron bioavailability represents a challenge for treating iron-related disorders. In this study, gastroresistance and iron intestinal absorption of an innovative granular formulation composed of ferric pyrophosphate, modified starch and phospholipids branded as Ferro Fosfosoma^®^ was investigated. Gastroresistant properties were studied using standard protocols, and a bioaccessible fraction was obtained by exposing a food supplement to in vitro digestion. This fraction was used for investigating iron absorption in Caco-2 and human follicle-associated intestinal epithelium (FAE) models. Ferro Fosfosoma^®^ showed an improved resistance to gastric digestion and higher intestinal absorption than ferric pyrophosphate salt used as a control in both models. In the FAE model, Ferro Fosfosoma^®^ induces larger iron absorption than in the Caco-2 monolayer, most likely due to the transcytosis ability of M cells. The larger iron absorption in the Ferro Fosfosoma^®^-treated FAE model corresponds to higher ferritin level, proving physiological iron handling that was once delivered by granular formulation. Finally, the formulation did not induce any alterations in viability and barrier integrity. To conclude, Ferro Fosfosoma^®^ favors iron absorption and ferritin expression, while preserving any adverse effects.

## 1. Introduction

Iron deficiency (ID) is one of the most widespread nutritional deficiencies in the world. People suffering from ID are mostly infants, young children, women with heavy menstruations and also pregnant and lactating women [1]. In normal healthy people, the main source of iron loss is through epithelial cell exfoliation from the intestine, skin and hair [2], and it accounts for about 0.5–2 mg per day [3]. Since iron is a micronutrient involved in many biological processes, a balance between iron uptake, transport, storage and metabolism is crucial to maintain iron homeostasis [4]. Despite iron being an essential trace element for the human organism, its non-physiological handling may cause toxicity due to iron’s ability to induce reactive oxygen species (ROS) and oxidative damage [5,6].

Alterations in dietary intake lead to iron dyshomeostasis and deficiency. To avoid iron deficiency, dietary supplementation is recommended [7]. The oral administration route is the most patient-compliant, safe and effective, so iron supplements based on ferrous or ferric salts are typically used for the treatment of ID. However, they are usually characterized by low bioavailability [8] and undesirable gastrointestinal side effects, such as abdominal pain, nausea, vomiting, diarrhea and constipation [9,10]. In these last years, innovative iron dietary supplements with new carriers have been developed to enhance iron intestinal absorption while reducing dosage and side effects [11]. For example, it has been demonstrated that liposomal iron encapsulation improves the efficiency of iron delivery with minimal side effects [12,13,14]. Moreover, an innovative formulation based on micronized microencapsulated ferric pyrophosphate has shown a higher intestinal bioavailability due to its small size and nanometer level of the particles that compose this formulation [15]. The efficacy of these technologies applied to different forms of iron supplements has been evaluated in several clinical trials [16,17,18].

In the present study, an innovative iron supplement formulation, named Ferro Fosfosoma^®^, composed of ferric pyrophosphate salt, modified starch, phosphatidylcholine (PC) and phosphatidyl-serine (PS) was tested. The chemical-modified starch used for this formulation was an acetylated starch, which is obtained by esterification of native starch with acetic anhydride [19], and it is widely used in a large variety of foods as a kind of thickener or stabilizer [20]. Enrichment with phospholipids is particularly interesting because this group of polar lipids act as enhancers of intestinal nutrient absorption. In fact, phospholipids in physiological conditions can form many kinds of assemblies, such as micelles and liposomes, triggering the release of poorly water-soluble molecules within the cells. To evaluate if this new supplement delivery system can improve iron intestinal absorption, a combination of an in vitro digestion process along with intestinal epithelium models was used to simulate digestion and absorption in the human intestine [21].

## 2. Results

### 2.1. Fe Content in Tested Formulations

The Iron (Fe) content in Ferro Fosfosoma^®^ and the control (ferric pyrophosphate salt) was analyzed by Inductively Coupled Plasma Mass Spectrometry (ICP-MS) (PerkinElmer Inc., Waltham, MA, USA) and was compared with the expected values. Ferro Fosfosoma^®^ contains 116 mg/g of Fe (11.6%), whereas ferric pyrophosphate salt contains 250 mg/g of Fe (25%). As depicted in Table 1, both measured Fe values were comparable with expected amount, and recovery was in the range of 80–110%.

### 2.2. Gastroresistance

Gastroresistance was assayed using protocols according to US pharmacopeia (USP). Briefly, equal amounts of Ferro Fosfosoma^®^ and control salt were dissolved in HCl 0.1 M and were incubated for 2 h at 37 °C. At the end of the incubation time, Fe titer was measured by ICP-AES analysis. As indicated in Table 2, after this treatment, the release of Fe from Ferro Fosfosoma^®^ was lower (3.4%) in comparison to that of the control preparation (7.5%).

### 2.3. Fe Intestinal Absorption

**Figure 1:**
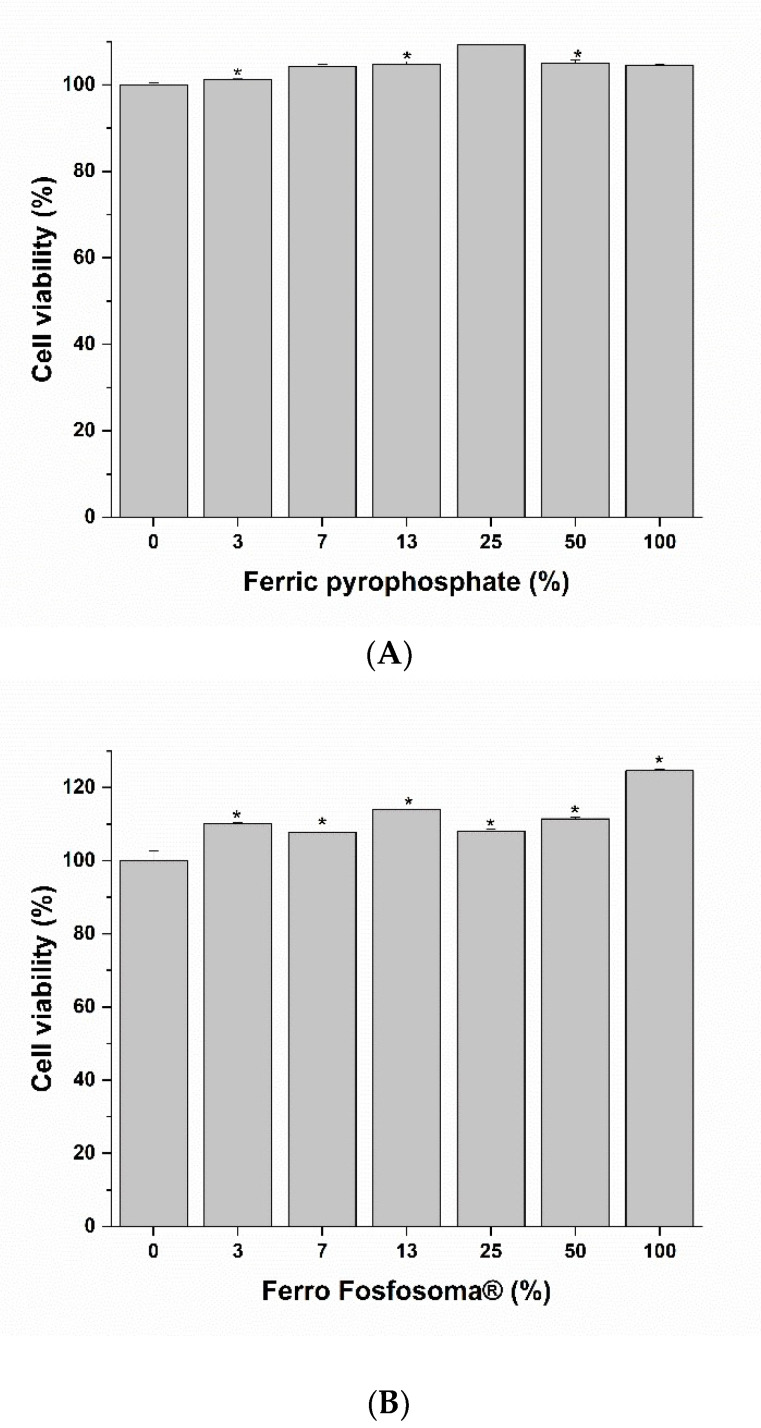
Effect on cell viability of Caco-2 cells. Cells were exposed to increasing concentrations of the bioaccessibile fractions of ferric pyrophosphate (**A**) and Ferro Fosfosoma^®^ (**B**) for 3 h, and then cell viability was determined using MTS assay. * *p* < 0.05.

**Figure 2:**
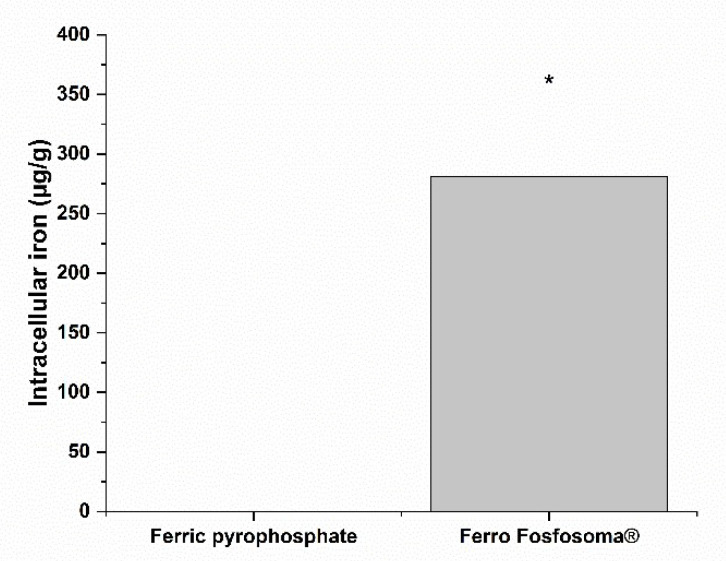
Intracellular Fe concentrations (μg/g cellular protein) in intestinal epithelial cell (Caco-2) monolayer at the end of the 3 h incubation with bioaccessible fractions of the two Fe formulations. * *p* < 0.05.

The bioaccessibility of dietary supplements refers to the active components released from its matrix in a stable form and available for absorption. To obtain an Fe bioaccessible fraction, an amount of Ferro Fosfosoma^®^ and control salt corresponding to 30 mg of Fe were subjected to an in vitro digestive process. After digestion, a complete fraction was collected to calculate the process recovery and supernatant corresponding to the bioaccessible fraction that was used for the absorption experiment. At the end of the in vitro digestion, in order to assess if this process could cause the loss of material, the amount of Fe was measured, and, as reported in Table 3, Fe recovery was close to 100% for both formulations. Fe that was released from the matrix was tested for intestinal absorption using the well-characterized in vitro Caco-2 model. Before performing the experiment, the impact of bioaccessible fractions of Ferro Fosfosoma^®^ and the control salt on intestinal mucosa viability was evaluated with the aim of correlating absorption to physiological routes instead of damages to barrier integrity. The Caco-2 monolayer was incubated for 3 h for increasing concentrations of bioaccessible fractions ranging from 0 to 100%. As indicated in Figure 1A,B, no reduction in Caco-2 viability was observed after 3 h of treatment at all tested concentrations; a slight increase in viable cells was found at higher concentrations, in particular after exposure to bioaccessible fractions of Ferro Fosfosoma^®^. Based on these results, bioaccessible fractions were directly used to determine intestinal Fe absorption by exposing a Caco-2 monolayer for 3 h. After incubation, intestinal Fe absorption was evaluated considering both intracellular and basolateral contents. Ferro Fosfosoma^®^ induces remarkable Fe absorption (281.12 ± 73.25 µg/g of cellular proteins) in comparison to the control formulation, which was lower than the limit of detection. (Figure 2 and Table 4).

Internalization of molecules through the intestinal barrier may occur by different routes [22,23]. Ferro Fosfosoma^®^ is characterized by the presence of two membrane phospholipids, such as phosphatidylserine and phosphatidylcholine, and Fe absorption can be mediated by transcytosis-a cellular transport due to vesicle internalization. To investigate the role of transcytosis in favoring the intestinal absorption of Fe delivered by Ferro Fosfosoma^®^, the human follicle-associated intestinal epithelium (FAE) model was used. This model was established by using a Caco-2/Raji B cells co-culture, since Raji B cells induce the transformation of a fraction of enterocytes into microfold (M) cells. This model is suitable for the investigation of intestinal absorption via transcytosis because M cells are specialized in the translocation of particles and macromolecules across the epithelial cell layer from the gut lumen to the lamina propria.

**Figure 3:**
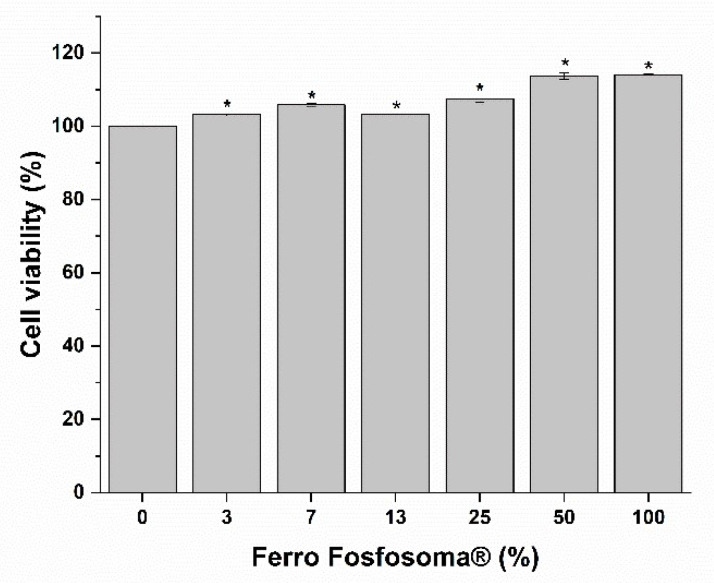
Effect on cell viability of human intestinal follicle-associated epithelium (FAE) monolayer. FAE monolayer was exposed to increasing concentrations of the bioaccessibile fractions of the granular form of ferric pyrophosphate for 3 h, and then cell viability was determined using MTS assay. * *p* < 0.05.

**Figure 4:**
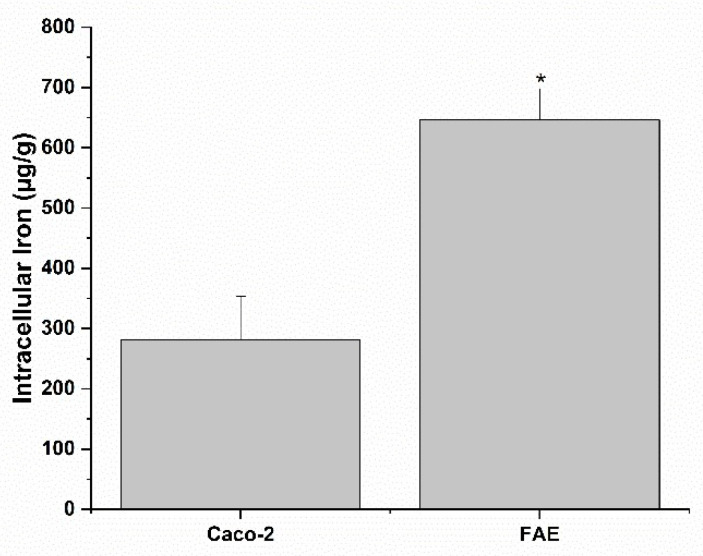
Intracellular Fe concentration (μg/g cellular proteins) in Caco-2 and human intestinal follicle-associated epithelium (FAE) monolayers at the end of the 3 h incubation with bioaccessible fractions of Ferro Fosfosoma^®^. * *p* < 0.05.

Moreover, in this case, no toxic effects were observed when exposing the FAE model to increasing concentrations of bioaccessible fractions of Ferro Fosfosoma^®^ for 3 h (Figure 3). Therefore, the FAE model was directly exposed to the highest concentration for 3 h, and internalized Fe was quantified by ICP-MS. The comparison of intestinal Fe absorption between Caco-2 and FAE models shows a higher absorption of Fe in the latter model (Figure 4 and Table 5), suggesting transcytosis as a relevant internalization route for Ferro Fosfosoma^®^.

Despite Fe absorption, the non-physiological handling of Fe by cells may lead to Fe deficiency and side effects. To evaluate the proper provision of Fe by the two formulations, intracellular ferritin levels were evaluated in the in vitro models, since they are involved in the Fe storage process, and their expression depends on intracellular Fe levels. As shown in Table 6, in the Caco-2 monolayer, Ferro Fosfosoma^®^ induces an increase in ferritin levels in comparison to the control. Moreover, ferritin amounts were further increased in the FAE model exposed to Ferro Fosfosoma^®^ in comparison to the Caco-2 monolayer (Table 7). Altogether, these findings suggest improved handling of the Fe delivered from Ferro Fosfosoma^®^ by intestinal epithelial cells in comparison to the control.

### 2.4. Impact of Fe-Based Formulations on Intestinal Epithelium

**Figure 5:**
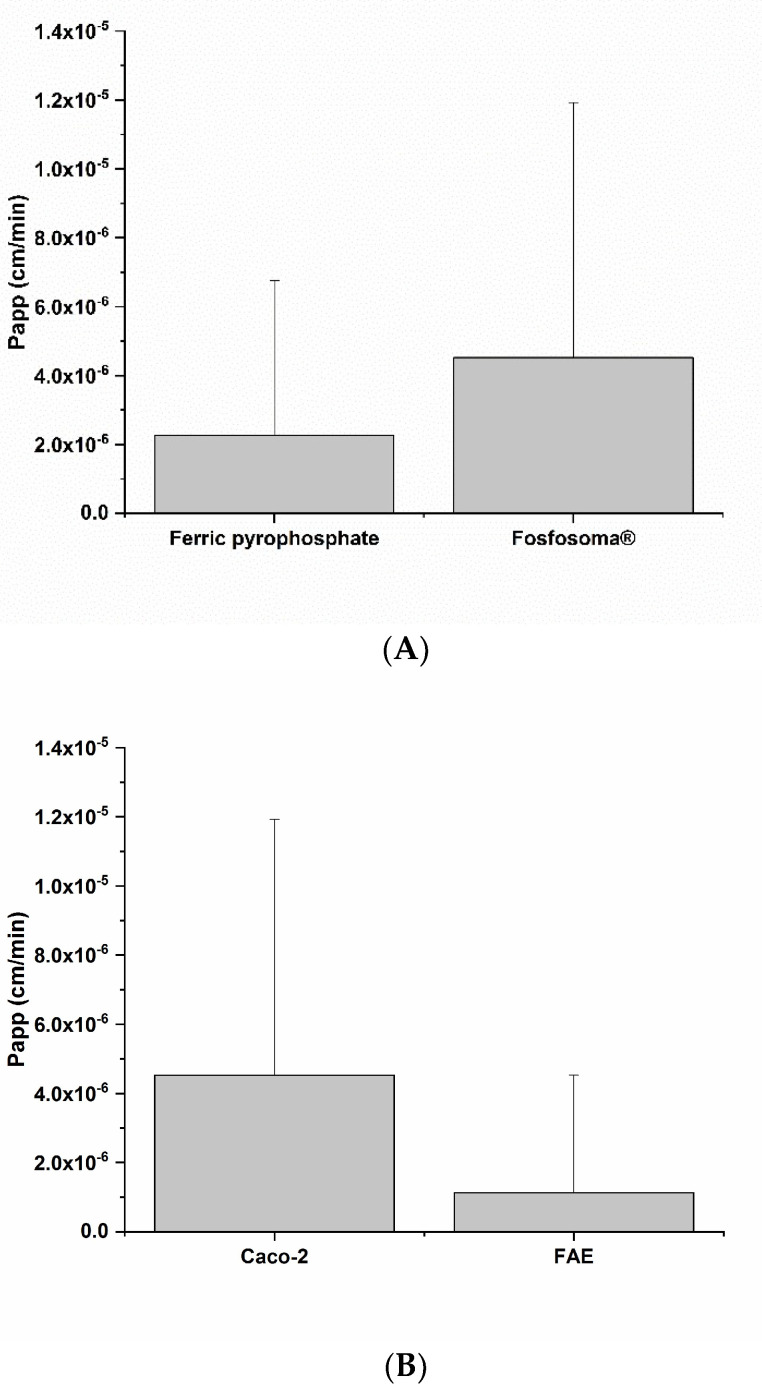
Impact of bioaccessible fractions of the two Fe formulations on apparent permeability (Papp) of Caco-2 cell monolayer model (**A**) after 3 h of incubation. Effect of Fosfosoma^®^ on Papp of Caco-2 cell monolayer and FAE models (**B**) after 3 h incubation. Intestinal epithelium integrity was further assessed by TEER measurement.

Aside from the benefits, Fe can cause oxidative damage, irritation and inflammation [24], so the safety of Fe-based dietary supplements needs to be carefully evaluated. The impact of tested Fe supplements on intestinal epithelium vitality and barrier integrity was evaluated. The impact on barrier integrity was evaluated by measuring the apparent permeability coefficient (Papp) and trans-epithelial electrical resistance (TEER). Bioaccessible fractions of Ferro Fosfosoma^®^ do not induce any significant increases in Papp in Caco-2 monolayers (Figure 5A). By comparing the Papp of the Caco-2 monolayer and FAE model exposed to Ferro Fosfosoma^®^, no differences were observed (Figure 5B).

As shown in Table 8, after 24 h incubation of the Caco-2 monolayer with bioaccessible fractions, the TEER values were similar to the results observed before the treatment. Moreover, in the FAE model, the TEER values after treatment for 24 h with bioaccessible fractions of Ferro Fosfosoma^®^ were comparable to those of the Caco-2 monolayer (Table 9).

## 3. Discussion

In the present study, Ferro Fosfosoma^®^, a new granular form of ferric pyrophosphate-based formulation containing modified starch, phosphatidylcholine (PC) and phosphatidylserine (PS), was studied regarding gastroresistance, intestinal absorption, iron functional value and safety. Ferro Fosfosoma^®^ exhibits a good higher gastroresistance than that of both Fe salt and sucrosomial Fe, a formulation in which ferric pyrophosphate is protected by a phospholipid bilayer plus a sucrester matrix. It has an enhanced intestinal absorption compared to that of the control formulation in the Caco-2 monolayer. Moreover, cellular Fe uptake was higher in the FAE model than it was in the Caco-2 monolayer. The FAE model represents M cells found in the Peyer’s patches, which are involved in intestinal transport from the apical surface to the basolateral surface of a variety of materials and immune system cells. This type of transcellular transport is known as transcytosis [25,26,27]. This finding reveals that Ferro Fosfosoma^®^ can exploit this alternative internalization route most likely due to the amphiphilic structure of the two phospholipids (PC and PS) present in the formulation, which may favor the interaction with the plasma membrane that is a crucial step for the intestinal transcellular transport of molecules. Finally, a good in vitro safety and tolerability profile was found.

## 4. Materials and Methods

### 4.1. Materials

Caco-2 human epithelial colorectal adenocarcinoma cells (ATCC^®^ HTB-37.) and human Burkitt’s lymphoma derived Raji cells (ATCC^®^ CCL-86.) were purchased from ATCC (Manassas, VA, USA). High glucose Dulbecco’s Modified Eagle Medium (DMEM), Hanks’ Balanced Salt Saline (HBSS), non-essential amino acids (NEAA), L-glutamine, Penicillin-Streptomycin mix, reagents for simulant digestive fluid preparation, Lucifer Yellow (LY) and an ELISA Kit specific for Human Ferritin were purchased from Sigma-Aldrich (St Louis, MO, USA). Fetal bovine serum (FBS) was purchased from Euroclone (Milan, Italy). Transwell^®^ insert was purchased from Millipore (Burlington, MA, USA). The CellTiter 96^®^ Aqueous One Solution Cell Proliferation Assay (MTS) was purchased from Promega (Madison, WI, USA). An external standard for ICP-MS external calibration (IQC-026) was purchased from Agilent (Santa Clara, CA, USA), and an internal standard (rodium) was purchased from Romil (Cambridge, UK).

### 4.2. Formulation Composition

Composition details of the granular form of ferric pyrophosphate (Ferro Fosfosoma^®^-, Neilos S.r.l., Naples, Italy) and the control (ferric pyrophosphate salt) are reported in Table 1.

### 4.3. Cell Cultures

#### 4.3.1. Caco-2 Cell Culture

The human epithelial colorectal adenormacinoma Caco-2 cells (passage 30 to 40) were maintained in a Caco-2 cell culture medium (Caco-2 CCM) (DMEM High Glucose medium supplemented with 10% FBS, 2% L-glutamine, 1% NEAA and 1% Penicillin-Streptomycin mix). The cells were grown in a controlled atmosphere incubator (85% relative humidity, 5% CO_2_ and 37 °C). Caco-2 cells were seeded at 2000 cell/cm^2^, and the medium was changed every other day. Cells were subcultivated by tryspinization every 7 d when they were 80–90% confluent.

#### 4.3.2. Raji Cell Culture

Human Burkitt’s lymphoma-derived Raji cells (passage 52 to 62) were maintained in a Caco-2 CCM. The cells were cultured at a density of 1 × 10^5^ cells/mL in a controlled atmosphere incubator (85% relative humidity, 5% CO_2_ and 37 °C) and were subcultured twice a week at a density of 1 × 10^5^ cell/mL.

### 4.4. Fe Content Determination in the Fe-Based Formulations

The Fe content of the Fe-based formulations was determined by inductively coupled plasma mass spectrometry. Briefly, a sample of Fe supplement equivalent to 300 mg was mineralized with nitric acid (1 mL) in a thermoblock at 70 °C for 8 h. If this process was not sufficient to completely digest the sample, complete mineralization was achieved with a microwave-based digestion system (MARS2, CEM Corporation, Matthews, NC, USA), with a two-step heating ramp. During the first step, the temperature was raised to 140 °C in 15 min, and it was held for 1 min. In the second step, the temperature was increased to 180 °C in 15 min, and it was held for 10 min. Once mineralized, the digested Fe supplement samples were transferred in pre-weighted polypropylene tubes and were diluted up to 50 mL, and the obtained solutions were weighed. Further dilution was applied when necessary. Fe (*m*/*z* = 53) content was determined with a NexIon 300D (PerkinElmer, Waltham, MA, USA), using external calibration and rodium as the internal standard. The measured amounts were compared to the declared ones, and Fe recovery was calculated as the percentage ratio between the measured and declared amount. LOD: 0.22 µg.

### 4.5. Determination of Gastroresistance of Fe-Based Formulation

To determine the gastroresistance of the formulation, a dissolution test was performed according to Unites States Pharmacopeia (USP) <711> for the dissolution, USP <2040> for disintegration and dissolution and USP <730> for ICP-AES analysis. The test was performed using 0.1 M HCl solution. Briefly, 1 g of sample was weighed in the vessel of the dissolution system filled with 900 mL of HCl 0.1 M. The dissolution was performed for 2 h at 37 °C and 75 rpm. After dissolution, 1 mL of the sample was diluted to 50 mL with HCl 0.1 M. Six replicates for each sample were prepared. The results are expressed as the percentage ratios between the Fe titer and the HCl 0.1 M volume.

### 4.6. Determination of Fe-Based Formulation Bioaccessibility

An amount corresponding to 30 mg of Fe (120 mg for ferric pyrophosphate salt and 258 mg for Ferro Fosfosoma^®^) was exposed to an in vitro digestion procedure, which was designed to simulate the physiological process in humans (i.e., oral, gastric and intestinal compartments). Briefly, all simulant digestive juices (i.e., saliva, gastric juice, duodenal juice and bile) were prepared according to [28] and were pre-heated to 37 °C. The digestion started by adding saliva (pH = 6.8 ± 0.1) to the formulation and by incubating the obtained bolus at 37 °C for 5 min under constant head-over-heels agitation to simulate the chewing phase. Subsequently, gastric juice (pH = 1.3 ± 0.1) was added to the bolus, and the pH was checked and, if necessary, was adjusted to 2.5 ± 0.5. The resulting chime was further incubated at 37 °C for 2 h under constant head-over-heels agitation to simulate gastric peristaltic movements.

In the next phase, duodenal juice (pH = 8.1 ± 0.1), bile (pH = 8.2 ± 0.1) and sodium bicarbonate solution were added. The pH of the obtained chyle was set at 6.5 ± 0.5, and it was rotated head-over-heels for another 2 h at 37 ° C. The digestive fluid volumetric ratio was strictly respected by adding saliva, gastric juice, duodenal juice, bile and sodium bicarbonate in the following ratio: 1:2:2:1:0.3. At the end of the digestive process, Fe concentrations in the complete digests were measured and compared to the applied levels in order to estimate the overall recovery of the process.

### 4.7. Intestinal Epithelium In Vitro Model

The intestinal and intracellular absorption of bioaccessible Fe fractions was determined using a human intestinal in vitro model based on a co-culture of Caco-2 and Raji cells, cultured as functional monolayers in Transwell^®^ inserts. Briefly, following the method described by Rieux et al., 2005 [29], Caco-2 cells (1.5 × 10^5^ cell/insert) were seeded in the apical compartment and were left to mature to functional enterocytes for two weeks. Apical (0.5 mL) and basolateral (1.5 mL) Caco-2 CCM was refreshed every other day. Following enterocyte maturation, Raji cells were added to the basolateral compartment (6.7 × 10^4^ cell/mL). Then, the co-culture was left to mature for another week to allow the differentiation of a fraction of the enterocyte population to microfold cell (M cell) . The medium of the cell culture was refreshed only apically with the same frequency as described before. Finally, prior to the Fe absorption experiment, Raji cells were removed, and the basolateral compartment was extensively washed. The resulting functional human follicle-associated epithelia (FAE) were characterized by polarized cells with morphological and functional aspects typical of enterocytes (i.e., microvilli presence, tight junctions and P-glycoprotein) and M cells (i.e., transcytosis process). Prior to the Fe absorption experiments, cell monolayer trans-electrical resistance (TEER) was measured with an epithelial volt/ohm meter (Millicell^®^ ERS2, Millipore Burlington, MA, USA) equipped with a chopstick sensor. Only FAE endowed with a TEER value > 300 W/cm^2^ were considered for the absorption experiments.

### 4.8. Evaluation of Fe Intestinal Absorption

The bioaccessible fractions were added to the apical compartment (1 mL) of the in vitro intestinal epithelium model, and the cell culture medium with 1% FBS was placed in the basolateral compartment (1.5 mL). After 1 and 3 h of incubation in a controlled atmosphere incubator (85% relative humidity, 5% CO_2_ and 37 °C) and under constant agitation (100 rpm) to simulate intestinal luminal shear stress, apical, basolateral and cellular (FAE epithelium) fractions were collected. The Fe content of the collected aliquots (apical, basolateral and cellular fractions) was determined by ICP-MS analysis.

### 4.9. Determination of Intracellular Ferritin

For intracellular ferritin determination, the protocol described by Scheers et al., 2014 [28] was followed. Briefly, after treatment with Fe-based formulations of bioaccessible fractions, FAE monolayers were washed multiple times to remove non-absorbed Fe and were left to incubate in modified Caco-2 CCM (1% FBS) for an additional 23 h (1 h exposure) and 21 h (3 h exposure) to allow for ferritin expression. At the incubation end, FAE-composing cells were detached by trypsinization, were pelleted and were sonicated in a lysis medium (0.1% of Triton X-100 in ddH_2_O supplemented with protease inhibitor cocktail). Finally, the ferritin content of the different cell lysates was determined by the ELISA (Enzyme-Linked ImmunoSorbent Assay) technique, using a commercially available kit. Ferritin levels were normalized for the total protein content of each sample, and the results are expressed as fold changes compared to the control.

### 4.10. Vitality and Barrier Integrity of the Intestinal Epithelium Model

Cell viability was evaluated by using an MTS assay, which is based on the reduction in MTS tetrazolium compound by viable cells to generate a colored formazan product that can be quantified by measuring the absorbance at 490 nm. Therefore, cell viability is directly proportional to absorbance. Vitality results are expressed as percentages (%) compared to digestive-fluid-treated FAE. Barrier integrity was evaluated by measuring both monolayer trans-epithelial electrical resistance (TEER) and apparent permeability (Papp). The TEER was determined by measuring FAE monolayer trans-epithelial electrical resistance before (pre-treatment), soon after (post-treatment) and following the 24 h recovery from treatment (24 h recovery). The TEER results are expressed, for each condition, as percentages (%) compared to the pre-treatment TEER values. Apparent permeability (Papp) was assessed by measuring Lucifer Yellow (LY) permeability. LY is a polar tracer used to investigate the paracellular permeability of a cell monolayer, since it is unable to pass through intact tight junctions. Briefly, 0.5 mL of 100 µg/mL LY in HBSS was added to the apical compartment of the intestinal epithelium in vitro model, and 1.5 mL of HBSS was placed in the basolateral compartment. Following 1 h of incubation in static conditions and a controlled atmosphere, the basolateral buffer was collected, and the permeated amount of LY was determined spectrofluorimetrically with a multiwell plate reader (428 nm excitation and 536 emission). The coefficient of permeability (Papp, cm/min) was calculated with the following formula:

Papp = (ΔC · V)/(Δt · A · C0)(1)

where ΔC/Δt is the flow of the molecule being transported across the monolayer during the incubation time (mM/min), V is the volume of the basolateral compartment (cm^3^), A is the area of the membrane (cm^2^) and C0 is the initial concentration of the molecule in the apical compartment. Apparent permeability results are expressed as cm/min.

### 4.11. Statistical Analysis

The results were statistically analyzed with a t-test, using OriginLab software (OriginLab Corporation, Northampton, MA, USA). The experiments were performed in triplicate, and the results are presented as the average ± standard deviation. A *p* value of ≤0.05 was considered significant.

## 5. Conclusions

The results of this study demonstrate that ferric pyrophosphate salt, enriched with modified starch and phospholipids PC and PS, exhibits improved resistance to acidic gastric environments and increased intestinal Fe absorption in comparison to the control formulation, and it is well-tolerated by intestinal epithelial cells and can represent a promising therapeutic strategy for Fe deficiency treatment.

## Figures and Tables

**Table 1:** Fe amount/dose in tested formulations, expressed as mg/g. The percentage of recovery was calculated as the ratio between measured and nominal Fe values. Results are expressed as mean ± standard deviation.

	Declared Amount (%)	Measured Amount (%)	Recovery (%)
**Ferric pyrophosphate**	30	25 ± 0.3	83.4 ± 1.4
**Ferro Fosfosoma^®^**	11	11.6 ± 0.1	105.8 ± 0.6

**Table 2:** Fe percentage after dissolution in HCl 0,1 M of the indicated formulations. Results are expressed as mean ± standard deviation.

	Ferric Pyrophosphate	Ferro Fosfosoma^®^
**Fe (%)**	7.5 ± 0.5	3.4 ± 0.2

**Table 3:** Percentage of Fe recovery of tested formulations at the end of in vitro digestive process. Results are expressed as mean ± standard deviation.

	Fe Expected Amount (mg)	Fe Measured Amount (mg)	Recovery (%)
**Ferric pyrophosphate**	30	29.3 ± 2.6	97.7 ± 8.7
**Ferro Fosfosoma^®^**	30	32.4 ± 0.4	108.1 ± 1.5

**Table 4:** Intracellular Fe levels in intestinal epithelial cells (Caco-2) at the end of the 3 h incubation with bioaccessible fractions of the two Fe formulations. Results are expressed as mean ± standard deviation. LOD 0.22 µg.

Fe Intestinal Absorption (µg/g)	
**Ferric pyrophosphate**	<LOD
**Ferro Fosfosoma^®^**	281.12 ± 73.25

**Table 5:** Intracellular Fe levels in Caco-2 and human intestinal follicle-associated epithelium (FAE) monolayers at the end of the 3 h incubation with bioaccessible fractions of Ferro Fosfosoma^®^. Results are expressed as mean ± standard deviation.

Fe Intestinal Absorption (µg/g)	
**Caco-2**	281.12 ± 73.25
**FAE**	646.22 ± 53.13

**Table 6:** Ferritin in Caco-2 cell monolayer exposed for 3 h to bioaccessible fractions of the two Fe formulations. Ferritin levels are expressed as fold changes using Ferric pyrophosphate salt as a reference.

Ferritin Level (Fold Change)	
**Ferric pyrophosphate**	1
**Ferro Fosfosoma^®^**	2.54

**Table 7:** Ferritin in Caco-2 cells and in human intestinal follicle-associated epithelium (FAE) monolayers exposed for 3 h to bioaccessible fractions of Ferro Fosfosoma^®^. Ferretin levels are expressed as fold changes using Caco-2 monolayer as a reference.

Ferritin Level (Fold Change)	
**Caco-2**	1
**FAE**	2.89

**Table 8:** TEER values in Caco-2 monolayer at the end of the 3 h incubation with bioaccessible fractions of ferric pyrophosphate salt and granular forms of ferric pyrophosphate. Results are expressed as mean ± standard deviation.

TEER (Ohms × cm^2^)		
	Pre-Treatment	Recovery 24 h
**Ferric pyrophosphate**	626.25 ± 70.88	604.58 ± 56.64
**Ferro Fosfosoma^®^**	660.8 ± 30.02	665.83 ± 16.64

**Table 9:** TEER values in Caco-2 and human intestinal follicle-associated epithelium (FAE) monolayers at the end of the 3 h incubation with bioaccessible fractions of Ferro Fosfosoma^®^. Results are expressed as mean ± standard deviation.

TEER (Ohms × cm^2^)		
	Pre-Treatment	Recovery 24 h
**Caco-2**	660.8 ± 30.02	665.83 ± 16.64
**FAE**	797.21 ± 35.25	634.58 ± 21.1

## Data Availability

Not applicable.
